# Fluorescein-stained confocal laser endomicroscopy versus conventional frozen section for intraoperative histopathological assessment of intracranial tumors

**DOI:** 10.1093/neuonc/noae006

**Published:** 2024-01-18

**Authors:** Arthur Wagner, Maria Charlotte Brielmaier, Charlotte Kampf, Lea Baumgart, Amir Kaywan Aftahy, Hanno S Meyer, Victoria Kehl, Julius Höhne, Karl-Michael Schebesch, Nils O Schmidt, Saida Zoubaa, Markus J Riemenschneider, Miriam Ratliff, Frederik Enders, Andreas von Deimling, Friederike Liesche-Starnecker, Claire Delbridge, Juergen Schlegel, Bernhard Meyer, Jens Gempt

**Affiliations:** Department of Neurosurgery, Klinikum rechts der Isar Technical University Munich School of Medicine, Munich, Germany; Department of Neurosurgery, Klinikum rechts der Isar Technical University Munich School of Medicine, Munich, Germany; Department of Neurosurgery, Klinikum rechts der Isar Technical University Munich School of Medicine, Munich, Germany; Department of Neurosurgery, Klinikum rechts der Isar Technical University Munich School of Medicine, Munich, Germany; Department of Neurosurgery, Klinikum rechts der Isar Technical University Munich School of Medicine, Munich, Germany; Department of Neurosurgery, Klinikum rechts der Isar Technical University Munich School of Medicine, Munich, Germany; Institute for AI and Informatics in Medicine & Muenchner Studienzentrum (MSZ), Technical University Munich School of Medicine, Munich, Germany; Department of Neurosurgery, Regensburg University Hospital, Regensburg, Germany; Department of Neurosurgery, Paracelsus Medical University, Nürnberg, Germany; Department of Neurosurgery, Regensburg University Hospital, Regensburg, Germany; Department of Neurosurgery, Paracelsus Medical University, Nürnberg, Germany; Department of Neurosurgery, Regensburg University Hospital, Regensburg, Germany; Department of Neuropathology, Regensburg University Hospital, Regensburg, Germany; Department of Neuropathology, Regensburg University Hospital, Regensburg, Germany; Department of Neurosurgery, University Hospital Mannheim, Mannheim, Germany; Department of Neurosurgery, University Hospital Mannheim, Mannheim, Germany; Department of Neuropathology, University Hospital Heidelberg and CCU Neuropathology, German Cancer Center (DKFZ), Heidelberg, Germany; Department of Neuropathology, Pathology, Medical Faculty, University Hospital Augsburg, Augsburg, Germany; Department of Neuropathology, Klinikum rechts der Isar Technical University Munich School of Medicine, Munich, Germany; Department of Neuropathology, Klinikum rechts der Isar Technical University Munich School of Medicine, Munich, Germany; Department of Neurosurgery, Klinikum rechts der Isar Technical University Munich School of Medicine, Munich, Germany; Department of Neurosurgery, Klinikum rechts der Isar Technical University Munich School of Medicine, Munich, Germany; Department of Neurosurgery, University Medical Center Hamburg-Eppendorf, Hamburg, Germany

**Keywords:** brain tumor histology, confocal laser endomicroscopy, frozen section, intraoperative diagnosis, telepathology

## Abstract

**Background:**

The aim of this clinical trial was to compare Fluorescein-stained intraoperative confocal laser endomicroscopy (CLE) of intracranial lesions and evaluation by a neuropathologist with routine intraoperative frozen section (FS) assessment by neuropathology.

**Methods:**

In this phase II noninferiority, prospective, multicenter, nonrandomized, off-label clinical trial (EudraCT: 2019-004512-58), patients above the age of 18 years with any intracranial lesion scheduled for elective resection were included. The diagnostic accuracies of both CLE and FS referenced with the final histopathological diagnosis were statistically compared in a noninferiority analysis, representing the primary endpoint. Secondary endpoints included the safety of the technique and time expedited for CLE and FS.

**Results:**

A total of 210 patients were included by 3 participating sites between November 2020 and June 2022. Most common entities were high-grade gliomas (37.9%), metastases (24.1%), and meningiomas (22.7%). A total of 6 serious adverse events in 4 (2%) patients were recorded. For the primary endpoint, the diagnostic accuracy for CLE was inferior with 0.87 versus 0.91 for FS, resulting in a difference of 0.04 (95% confidence interval −0.10; 0.02; *P* = .367). The median time expedited until intraoperative diagnosis was 3 minutes for CLE and 27 minutes for FS, with a mean difference of 27.5 minutes (standard deviation 14.5; *P* < .001).

**Conclusions:**

CLE allowed for a safe and time-effective intraoperative histological diagnosis with a diagnostic accuracy of 87% across all intracranial entities included. The technique achieved histological assessments in real time with a 10-fold reduction of processing time compared to FS, which may invariably impact surgical strategy on the fly.

**Key Points**

- Confocal laser endomicroscopy (CLE) allows for real-time intraoperative assessment of tumor histology in vivo.
- First clinical trial investigating its accuracy for intracranial tumors.
- Reaching an accuracy of 87%, CLE drastically expedited surgical workflow.

**Importance of the Study**

Alongside tumor mass reduction, neuro-oncological care is founded on the identification of the tumor entity. Currently, an intraoperative diagnosis can only be achieved by frozen section (FS), which is time-consuming and traumatic. A viable alternative has recently been explored in several oncological specialties with confocal laser endomicroscopy (CLE) that allows for a real-time in vivo histological assessment without traumatizing tissue. Our trial is the first and largest clinical investigation comparing the diagnostic validity of CLE with routine FS for intracranial mass lesions, accomplishing an accuracy of 87% referenced with the final histopathological assessment. Compared to routine FS, the novel technique results in a 10-fold reduction of time expedited until an intraoperative diagnosis is made through telepathology consultation. The surgeon is thus provided with real-time feedback as to the tumor entity, allowing them to adapt their surgical strategy on the fly and maximize the outcome for the patient.

In neurosurgery, intraoperative histopathological frozen section (FS) analysis represents a crucial tool for the rapid assessment of tumor entity and the achievement of tumor-free resection margins. The method, however, remains rather time-consuming, sometimes prolonging surgical times and demonstrating diagnostic accuracy reaching as low as 78.4% in comparison with the final histopathology, depending on the technique used.^1,2^ To combat these shortcomings, another promising adjunct was developed in the form of intraoperative Fluorescein-stained confocal laser endomicroscopy (CLE), for which there has been little scientific evidence so far. To date, only a few articles have explored its utility and general applicability predominantly in ex vivo models or uncontrolled case series of various entities.^3–6^ In principle, the technique allows for histopathological assessment of tumor tissue in vivo without the need for fixation and transport of the sampled tissue, aiming for a so-called in situ *digital biopsy* and thereby substantially improving operative precision and surgical times.^3,7,8^ Combined with Fluorescein sodium as a contrast-enhancing intraoperative fluorescent stain, the operating surgeon may then inspect lesional tissue firsthand, evaluate it for tumorous cells in real time, and adjust their resection strategy in a more immediate fashion.^8–10^

This clinical trial aimed to demonstrate that CLE evaluation by a neuropathologist is not inferior to routine FS analysis for the histopathological assessment of lesional tissue during intracranial surgery.

## Methods

### Patient Population

At 3 participating sites (Department of Neurosurgery, Klinikum rechts der Isar, Technical University Munich; Department of Neurosurgery, University Hospital Regensburg; and Department of Neurosurgery, University Hospital Mannheim), every patient aged >18 years undergoing elective surgery for the removal of an intracranial lesion was screened. Cases were not preselected based on the prospective entity of the lesion, and every intracranial mass was eligible. Patients unable to provide written informed consent were excluded from the trial. Patients with a known allergy to Fluorescein sodium, liver disease in stage Child B or C, or renal dysfunction were not permitted for trial inclusion since Fluorescein sodium is metabolized by glucuronidation and excreted via the kidneys within 48–72 hours. Thus, patients under medication with beta-blockers, digoxin, quinidine, probenecid, and inhibitors of glucuronidation, such as immunosuppressants, were excluded from trial participation when the medication could not be discontinued temporarily.

### CLE Setup

For this trial, the Zeiss CONVIVO CLE device was employed for all cases at all sites. It consists of a CLE probe connected to a user station fitted with a touchscreen for viewing the microscopy images intraoperatively and setting up image parameters during usage. The field of view amounts to 475 × 267 µm with a screen resolution of 1920 × 1080 pixels. The small, blunt, and versatile CLE probe resembles an instrument that is held in one hand and inserted without traumatizing healthy tissue, comparable with a standard suction device (see Supplementary Figure 1). The complete setup, including the CLE probe and its sterile drape, is CE-certified and approved for routine clinical use in the participating centers. Through a network port, the device allows for transmission of a digital live feed from the probe, accessible via a remote workplace for neuropathological consultation.

### CLE Procedure

**Figure 1.**
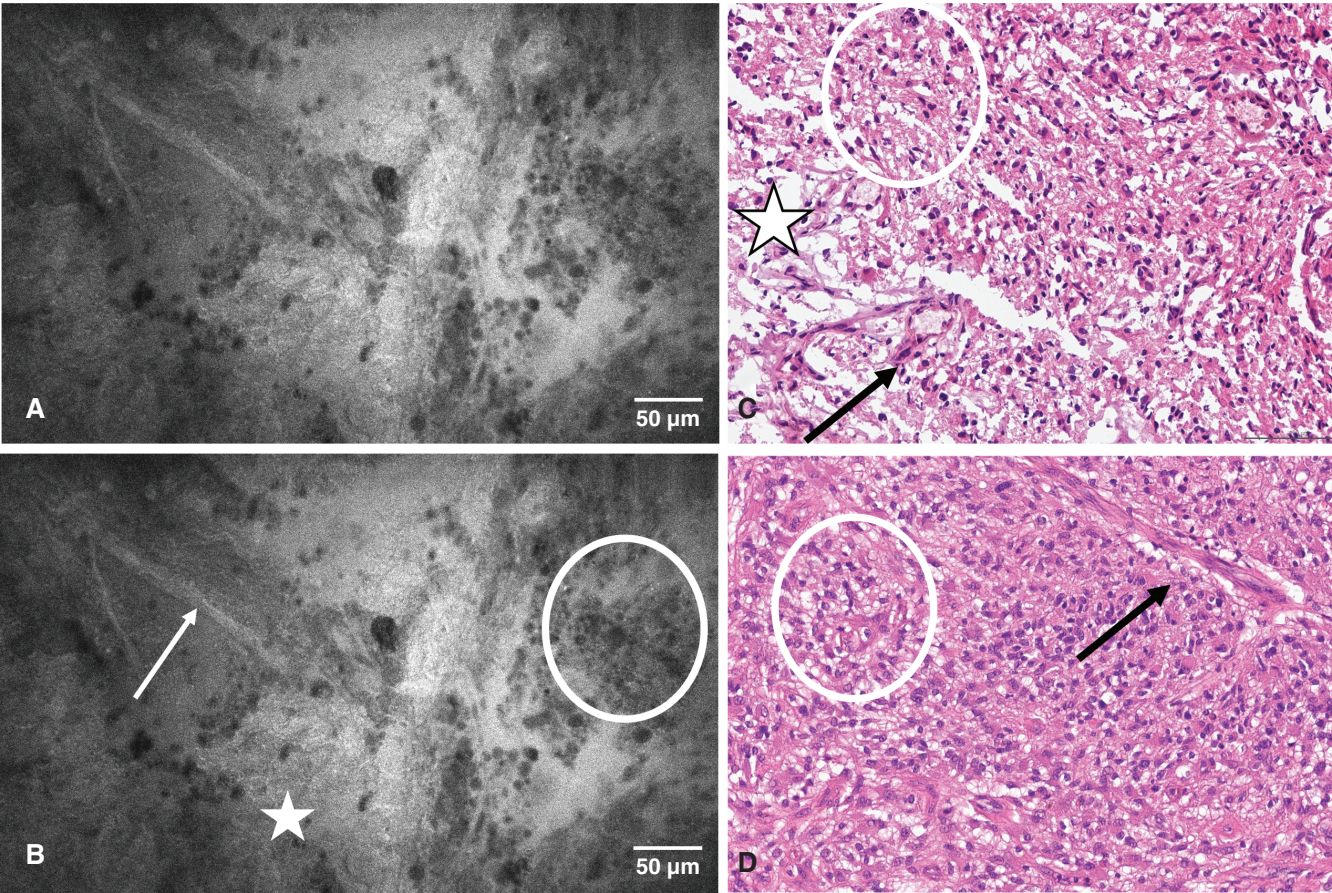
CLE image unmarked (A) and marked (B), hematoxylin–eosin stains of FS (C), and final histopathology smear (D) of a 74-year-old female with a right paratrigonal IDH wild-type glioblastoma. The CLE image is concordant with hallmark histological features of a glioblastoma, with necrotic areas (stars), pleomorphic glial tumor cells with hyperchromatic nuclei (circles), and vascular proliferates (arrows). Abbreviations: CLE, confocal laser endomicroscopy; FS, frozen section; IDH, isocitrate dehydrogenase.

**Figure 2.**
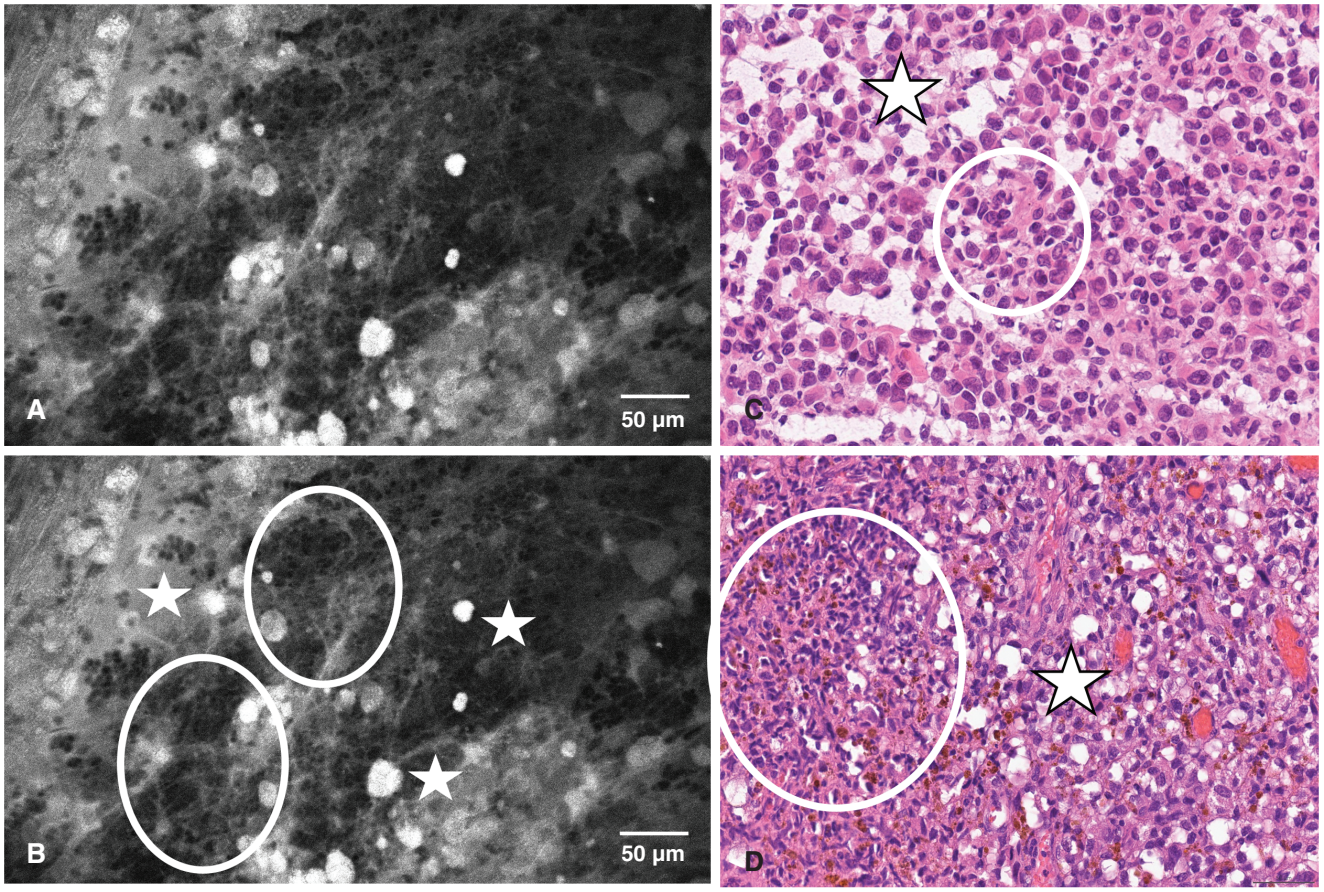
CLE image unmarked (A) and marked (B), hematoxylin–eosin stains of FS (C), and final histopathology smear (D) of a 68-year-old female with a left temporal melanoma metastasis, symptomatic with grand-mal seizures. The CLE demonstrates spots of auto-fluorescence (stars) as well as denser cellular areas (circles). FS assessment was ambivalent between melanoma and glioma, rendering the in vivo CLE the more conclusive diagnosis in this case. Abbreviations: CLE, confocal laser endomicroscopy; FS, frozen section.

**Figure 3.**
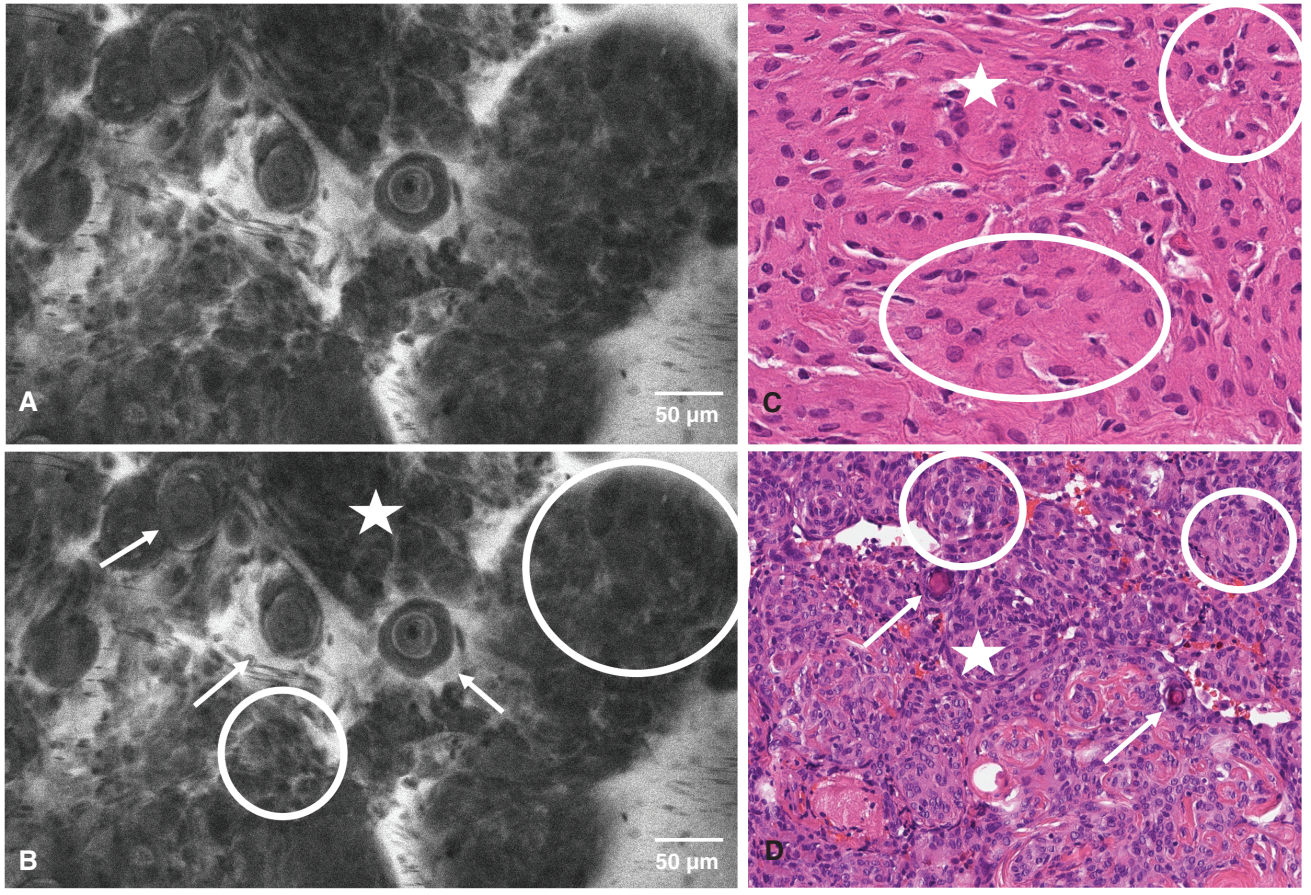
CLE image unmarked (A) and marked (B), hematoxylin–eosin stains of FS (C), and final histopathology smear (D) of a 68-year-old female with a sphenoidal plane meningioma. The transitional meningioma features both meningothelial (lobular, pseudo-syncytial growth; circles) and fibrous phenotypes (spindle-shaped cells, collagen fibers; stars) with a high number of psammoma bodies (arrows). Abbreviations: CLE, confocal laser endomicroscopy; FS, frozen section.

Fluorescein sodium was administered intravenously with a bolus injection of 5 mg/kg of bodyweight 20–40 minutes prior to the planned tumor resection. After the preparation of the lesion to be resected, the CLE probe was draped and positioned on the surface of presumed tumor tissue, aided by neuronavigation to direct the probe at a representative tissue specimen. A stack of 5 images was recorded for neuropathological assessment (Figures 1–3). The identical specimen was then biopsied with a grasping forceps for FS analysis. The FS specimen was labeled according to its anatomical location, and immediately sent for histopathological workup according to the procedural standards of each participating site. A digital stopwatch was used to log the time expedited from draping the CLE probe to recording the last stack of images and the time elapsed from removing the tissue for FS analysis to receiving the result via phone call. Essentially, the FS workflow represented the standard of care for the respective site. The remaining course of the surgery was unaltered.

### Pathological Analysis

The digital projection of the CLE imaging was evaluated centrally by a trained neuropathologist (J.S.) for all participating centers after surgery. The evaluation focused on diagnosing the entity of the investigated lesional tissue. FS assessments were performed by individual neuropathologists according to the routine conduct of the respective site. The neuropathologists received the same clinical case information both with the CLE images and the FS specimens. No cross-evaluation was allowed between the 2 techniques, and the neuropathologists did not consult each other during the assessment. For the CLE assessment, the diagnoses were stratified into “metastasis,” “low-grade glioma” (LGG; ie, diffuse astrocytoma World Health Organization Central Nervous System [WHO CNS] grade 2), “high-grade glioma” (HGG; ie, anaplastic astrocytoma WHO CNS grade 3, glioblastoma WHO CNS grade 4), “meningioma,” “schwannoma,” “ependymoma,” “reactive inflammation,” or “others” (including iron deposits, unspecified lesions, and unknown entities).

### Endpoint and Safety Analyses

For the primary endpoint, the diagnostic accuracy of the CLE assessment of tumor tissue was compared against FS analysis, the current gold standard. Accuracy was defined as the number of correctly classified diagnoses divided by the total number of assessments for each method, respectively. The final histopathological analysis was used as a reference for both methods separately. The diagnosis by the respective method was only rated “correct” when it exactly corresponded with the conventional white-light microscopy with immunohistochemistry. The concordance between the preliminary and definitive results was set as a dichotomous assessment (“yes” or “no”). The primary endpoint constituted demonstrating noninferiority of CLE to FS analysis, while noninferiority was defined as statistically equivalent accuracy rates for both. Thus, noninferiority was predefined in the case where the absolute difference in accuracies of the methods did not exceed 5%.

Secondary analyses included the safety assessment by documentation of adverse events (AEs) and serious AEs (SAEs) during surgery and until the end of follow-up. AEs were treated according to the protocol of the respective site. The follow-up concluded with the availability of the final histopathological result. The classification of all AEs followed the Medical Dictionary for Regulatory Activities (MedDRA) with System Organ Class and Preferred Terms. SAE severity was classified according to the Common Terminology Criteria for Adverse Events (CTCAE).

A series of post hoc analyses exceeding the originally planned analysis laid out in the study protocol were carried out to explore the CLE technique’s interrater concordance and capacity to discriminate between tumorous and nontumorous entities. Fleiss Kappa statistics and cross-tabulation for sensitivity and specificity after dichotomization into *Tumor* including the primary categories *HGG*, *LGG*, *Metastasis*, *Meningioma*, *Schwannoma*, and *Ependymoma*, and *Nontumor* including *Reactive*, *Inflammation*, and *other* were thus appended to the statistical analysis protocol and included as Supplementary Material.

### Ethical Considerations

All procedures were indicated and conducted in compliance with our department’s standards and the Declaration of Helsinki. The Ethics Committee Klinikum rechts der Isar of the Technical University Munich (Ethikkommission Klinikum rechts der Isar der Technischen Universität München) granted a positive vote (Reference 75/20 Af-KK). The trial had been registered in the European Clinical Trials Database (EudraCT: 2019-004512-58) before initiation. Written informed consent was obtained from every individual before study participation.

## Results

### Epidemiology

In total, 210 patients were included in this trial between November 2020 and June 2022. The full analysis set (FAS) consisted of 203 patients. Reasons for exclusion from the FAS were withdrawal of consent before surgery (*n* = 2, 1.0%), technical defect of the CLE device (*n* = 2, 1.0%), and lack of FS results due to deficient quality of the specimens (*n* = 3, 1.4%). Most patients were recruited in Munich (*n* = 117, 57.6%), followed by Regensburg (*n* = 69, 34.0%) and Mannheim (*n* = 17, 8.4%). The baseline characteristics of the cohort are depicted in Table 1. With a mean weight of 75.9 kg (standard deviation [SD] 17.3 kg), a mean Fluorescein dose of 379.43 mg (SD 87.43 mg) was administered.

**Table 1.** Demographics and Baseline Characteristics

	*N* = 203
Sex, *n* (%)	
Female	112 (55.2%)
Male	91 (44.8%)
Age group, *n* (%)	
Adults (18–64 years)	119 (58.6%)
Elderly (65–84 years)	81 (39.9%)
Senior (85 years and over)	3 (1.5%)
Age (years)	
Mean	58.1
Std	15.9
Min	22
Median	61
Max	87
Number of ongoing comorbidities (median [min–max])	1 [0–8]
Previous intracranial surgeries, *n* (%)	42 (20.0%)
Previous intracranial treatment, *n* (%)	47 (22.4%)
Recurrence, *n* (%)	33 (15.7%)

Abbreviations: Max, maximum; Min, minimum; Std, standard deviation.

The final histopathological analysis resulted in 49 (24.1%) metastases, 9 LGGs (4.4%), 77 (37.9%) HGGs, 46 (22.7%) meningiomas, 4 (2.0%) schwannomas, 2 (1.0%) ependymomas, 3 (1.5%) reactive lesions, 1 (0.5%) inflammatory processes, and 12 (5.9%) lesions classified as “other.”

### Medical History and Comorbidities

Most patients exhibited at least 1 concomitant disease (*n* = 147, 72.4%). The median number of concomitant diseases per patient was 1 and ranged from 0 to 8. In the FAS, 42/203 (20.0%) patients had previous intracranial surgeries. Other than surgery, 47 patients underwent previous intracranial treatment such as irradiation or chemotherapy (22.4%). A total of 33 patients underwent surgery for recurrence (15.7%).

### Adverse Events

A total of 121 AEs were reported in 57 (28%) patients (Table 2). Overall, 63/121 (52%) AEs were deemed to be possibly related to the CLE, and 61/121 (50%) of the AEs were rated as possibly related to Fluorescein. Moreover, 75/121 (62%) AEs were rated as grade 1 (mild); 42/121 (35%), grade 2 (moderate); 2/121 (2%), grade 3 (severe); 0%, grade 4 (life-threatening); and 2/121 (2%), grade 5 (fatal). None of the AEs graded 3–5 were rated as related to the CLE device or Fluorescein (Supplementary Table 4).

**Table 2.** Adverse Events (AEs) and Serious Adverse Events (SAEs)

		Subjects Affected	Severity Grade (CTCAE), Events				
	Events	*n* (%)	1	2	3	4	5
Total AEs	121	57 (28)					
Total SAEs	6	4 (2)	0	3	1	0	2
Infections and infestations	2	1 (0)	—	2	—	—	—
CNS ventriculitis	1	1 (0)	—	1	—	—	—
Meningitis	1	1 (0)	—	1	—	—	—
Nervous system disorders	4	3 (1)	—	1	1	—	2
Brain edema	1	1 (0)	—	—	1	—	—
Cerebral hemorrhage	1	1 (0)	—	—	—	—	1
Cerebral infarction	1	1 (0)	—	—	—	—	1
Epilepsy	1	1 (0)	—	1	—	—	—

Abbreviation: CTCAE, Common Terminology Criteria for Adverse Events.

### Serious Adverse Events

A total of 6 SAEs in 4 (2%) patients were recorded in the trial database. Table 2 summarizes all SAEs using the MedDRA System Organ Class and Preferred Terms. SAE severity was classified according to CTCAE. One patient with 2 SAEs died during the trial conduct due to a cerebral hemorrhage with infarction. The other 4 SAEs resolved fully without sequelae. The number and severity of AEs and SAEs were consistent with the patient population, and none of the SAEs were related to either the CLE or Fluorescein.

### Efficacy Results

#### Primary endpoint.—

The accuracy of the CLE referenced with the final histopathological diagnosis was 0.87 (176/203) on the FAS, whereas the accuracy of the FS analysis referenced with the final histopathological diagnosis was 0.91 (184/203) on the FAS (Table 3). The difference between the methods’ accuracies was −0.04 with a 95% CI (−0.10; 0.02), which misses noninferiority (*P* = .367). The concordance between CLE and FS diagnoses was 0.76. FS provided correct diagnoses, while CLE was incorrect in 24 of 185 cases (13.0%). The opposite, a correct diagnosis by CLE in contrast to FS, was achieved in 16 of 177 cases (9.0%), which resulted in no statistically significant difference (*P* = .724).

**Table 3.** Accuracies of the CLE and FS Methods Referenced With the Final Histopathological Diagnosis

		Final Histopathology									
		Metastasis	LGG	HGG	Meningioma	Schwannoma	Ependymoma	Reactive	Inflammation	Other	Sum
CLE	Metastasis	42	0	2	0	0	0	2	0	3	49
	LGG	0	5	1	0	0	0	0	0	3	9
	HGG	6	3	73	1	0	0	0	0	1	84
	Meningioma	0	0	1	44	1	0	0	0	0	46
	Schwannoma	0	0	0	1	3	0	0	0	0	4
	Ependymoma	0	0	0	0	0	2	0	0	0	2
	Reactive	0	0	0	0	0	0	1	0	0	1
	Inflammation	0	0	0	0	0	0	0	1	0	1
	Other	1	1	0	0	0	0	0	0	5	7
	Sum	49	9	77	46	4	2	3	1	12	203
FS	Metastasis	41	0	1	1	0	0	0	0	0	43
	LGG	0	7	0	0	0	0	0	1	0	8
	HGG	0	0	72	0	0	0	0	0	0	72
	Meningioma	0	0	0	45	0	1	0	0	0	46
	Schwannoma	0	0	0	0	4	0	0	0	0	4
	Ependymoma	0	0	0	0	0	1	0	0	0	1
	Reactive	0	1	2	0	0	0	2	0	0	5
	Inflammation	0	0	0	0	0	0	0	0	0	0
	Other	8	1	2	0	0	0	1	0	12	24
	Sum	49	9	77	46	4	2	3	1	12	203

Abbreviations: CLE, confocal laser endomicroscopy; FS, frozen section; HGG, high-grade glioma; LGG, low-grade glioma. Orange hue denotes “correct” diagnosis of respective method in concordance with final histopathology; blue hue denotes “incorrect” diagnosis.

#### Secondary endpoints.—

In the trial, the mean duration of surgery was 189 (SD 79) minutes. The CLE assessment time was a median of 3 (range, 1–15) minutes, whereas the duration of the FS assessment was a median of 27 (range, 10–110) minutes. The Wilcoxon signed-rank test for related samples comparing the duration of the 2 assessments was statistically significant, with a mean difference of 27.5 minutes (SD 14.5; *P* < .001). A cutoff of 10 minutes was used to classify the duration in “<10 minutes” or “≥10 minutes.” Only 1 CLE procedure lasted >10 minutes, whereas all FS analyses lasted >10 minutes (*P* < .001).

#### Post hoc analyses.—

In the post hoc analyses, the accuracies for both CLE and FS were stratified by the most common tumor entities. CLE achieved accuracies of 0.96 for LGGs, 0.93 for HGGs, 0.98 for meningiomas, 0.93 for metastases, and a mean accuracy of 0.95 across all tumor entities combined. FS achieved accuracies of 0.99 for LGGs, 0.98 for HGGs, 0.99 for meningioma, 0.95 for metastasis, and 0.98 across all tumor entities combined, resulting in a mean difference of accuracies of −0.03 (95% CI −0.06; 0.01) for tumor entities. In the meningioma subgroup analysis, the accuracies of CLE and FS were statistically equivalent under consideration of the 5% noninferiority threshold.

The sensitivities and specificities of CLE were 0.56 and 0.99 for LGGs, 0.86 and 0.95 for HGGs, 0.98 and 0.99 for meningiomas, and 0.86 and 0.95 for metastasis, respectively. The sensitivities and specificities of FS were 0.78 and 0.99 for LGGs, 0.94 and 1.0 for HGGs, 0.96 and 0.99 for meningiomas, and 0.84 and 0.99 for metastasis.

A post hoc analysis of a blinded assessment by 2 neuropathologists not involved in the primary assessment resulted in accuracies of 0.85 (173/203; 95% CI 0.80–0.90) for rater CD and 0.84 (170/203; 95% CI 0.78–0.89) for rater FLS, referenced with the final histopathology. The interrater agreement across all entities amounted to a Fleiss Kappa of 0.872 (95% CI 0.826–0.918; see Supplementary Table 1). When stratified by entity, tumor entities in general demonstrated high agreement coefficients, while the nontumor rating categories exhibited poor interrater agreement (see Supplementary Table 2). The CLE technique’s sensitivity and specificity to discriminate between tumorous and nontumorous tissue were analyzed post hoc by dichotomization and cross-tabulation; overall, a CLE rating corresponding to any tumor category had a sensitivity of 98.9%, whereas the specificity amounted to 43.8% (see Supplementary Table 3).

## Discussion

### CLE Procedure and Diagnostic Accuracy

Recently, it has been of emerging academic and clinical interest to accommodate the shortcomings of FS analysis with the development of new appliances. A glaring detriment of said assessment traditionally lies in their time-consuming and inherently *invasive* nature, requiring tissue to be extracted and transferred for sampling, embedding, processing, and lastly interpreting. These steps consume valuable intraoperative time, and it is not unusual for the interpreting neuropathologist to report inconclusive information because of insufficient tissue quality or uncertainty as to the origin of the material within the boundaries of the surgical site. The solution to these detriments prompted a desire to microscopically assess intravital tissue in situ in *real time* by endomicroscopy, which was incorporated into an end-user solution by several suppliers.

The CLE procedure is facilitated by a handheld, blunt probe, that is inserted into the surgical site without traumatizing healthy tissue.^9,11,12^ Combined with fluorescent staining of tissue with Fluorescein sodium, intravital tissue is visualized on a single-cell level, where the dye serves as a contrast enhancer (Figures 1–3). In recent investigations, Fluorescein sodium has emerged as a promising agent for the intraoperative fluorescent staining of cerebral metastases.^13,14^ A recent study showed accumulation of this fluorescent dye in highly vascularized tissue such as malignant cerebral lesions.^14^ Naturally, the application of Fluorescein begets a certain risk for AEs and SAEs, as elucidated in our results. While the rates of 28% for AEs and 2% for SAEs are substantial, the morbidity of our recruited population consisting predominantly of patients with malignant intracranial disease must be factored into this consideration. It is difficult to discern postoperative complications from true AEs related to the study procedures, specifically in a neurosurgical patient population that is notoriously vulnerable, although our reported AE and particularly SAE rates appear to be congruent with our previous series on complications in a comparable cohort.^15^ Further, the AEs that were rated as possibly related to the CLE procedure or Fluorescein administration are known as common side effects of Fluorescein. Paired with the low invasiveness of the CLE probe, we assume that the placement of the probe itself does not bear any immediate risks when handled properly, which corroborates the need to evaluate the administration of Fluorescein on an individual basis in lieu of a better-suited fluorophore at present.

This study represents the first and largest multicenter clinical trial investigating the accuracy of CLE compared with conventional FS. Another clinical trial with a similar design is underway (Bern, Switzerland, CLEBT, NCT04280952). We focused on the concordance of the histopathological diagnoses acquired by CLE with the definitive histopathological assessment in an unselected population of patients with an intracranial lesion to reflect real-life conditions. The accuracy that was achieved with CLE was only slightly surpassed by FS, yet it constitutes a servicable mark per se. The statistically inferior accuracy of 4% for CLE in light of a predefined noninferiority threshold of 5% within our trial protocol must be perceived in the context of (1) the range of entities examined; (2) the learning curve a novel technique such as CLE is very much still governed by; and (3) the relatively high accuracy that has been reached with the routinely conducted gold standard FS. It must be noted that CLE, as an adjunct conceived for intracranial tumor assessment, at present certainly reaches its limits with the assessment of less well-circumscribed lesions such as reactive processes, inflammatory, and nonneoplastic lesions. This detriment must at present in part also be seen second to a lack of expertise with these lesions, but moreso that they may be beyond the primary scope of the technique. Naturally, the assessment of these *exotic* entities is subject to improvement over time and experience gathered with the technique, if only to better delineate them from the entities of primary interest, such as gliomas, meningiomas, and metastases. This is reflected in the favorable results of our overall and entity-specific sensitivities and specificities, with a diagnostic accuracy equivalent to FS for meningiomas in particular, but hampered by the low specificity to differentiate between tumorous and nontumorous tissue in the post hoc analyses. One may conclude that CLE thus confirms a tumor diagnosis when the clinical context provided the assumption initially but does not achieve a secure rule-out of tumor tissue in ambiguous cases, although this notion stems from a somewhat unbalanced analysis owing to the scarcity of said ambiguous cases in comparison to tumors in our cohort.

Lastly, the FS technique reaches diagnostic accuracies between 85% and 90% in older retrospective series, which is well below our figure and arguably thwarts statistical noninferiority in our trial.^16–18^ Unsurprisingly, the highest discrepancy is found for entities with less distinctive histological features, such as diffuse astrocytomas and oligodendrogliomas, a drawback that both CLE and FS share.

### Surgical Workflow and Value of Real-Time Pathological Assessment

During the trial, it became evident that, moreso than with the FS analysis, the quality and aptitude of the CLE images are contingent on the surgeon’s application of the device. Not unlike the selection of a suitable and representative sample for FS, the tissue used for CLE assessment must be chosen with consideration. In turn, however, the surgeon receives immediate feedback on the quality of the recorded imaging and may adapt the positioning of the probe on the fly or scan a different locale in search of a more characteristic assortment of cells, without traumatizing the tissue and needing to wait for the transport and procession of the sample. CLE boasts a valuable asset herein, since now a conclusive diagnosis can be made in tandem with the digitally consulting neuropathologist who can appraise the images in real time—coining the newly conceived domain of *telepathology*.^8,19,20^ The implications of these considerations are mirrored in our abridged assessment times for CLE, accomplishing an almost 10-fold reduction in processing time compared with conventional FS, and through familiarization with the workflow, this advantage will possibly be exacerbated further.^21^

The determination of the entity in situ immediately informs the surgeon’s strategy for a given case. The absence of hallmark clinical and imaging features that are available in the preoperative setting may greatly impede on the surgical approach in consideration of distinctly differing strategies and goals HGGs and metastatic lesions entail, for instance.^22,23^ Similarly, the distinction between tumorous and nontumorous lesions requires a representable sample of macroscopically oftentimes non too conspicuous tissue. Failure to secure suitable tissue may render FS ineffectual and time costly, regularly necessitating extraction of another sample.

The possible applications of CLE extend beyond the mere intraoperative diagnosis. Oncological surgery is formulated on the principles of tumor volume reduction without impairment of functional integrity and profiling of the tumor through histomorphological and molecular characteristics.^24–27^ In this respect, intracranial lesions reprise a distinct role for various reasons; predominantly, it is oftentimes difficult to discern tumorous from surrounding healthy tissue under conventional white-light microscopy, and surgical resection usually represents the first encounter with the lesion’s histology within the oncological therapeutic regimen.^8,23,28^ Thus, it has long been of primary interest to improve the intraoperative distinction from eloquent areas and visualization of tumor margins with the aid of fluorescent dyes, neuronavigation, intraoperative ultrasonography, preoperative functional mapping, and intraoperative magnetic resonance imaging.^26,29–36^ This spectrum of surgical auxiliaries may be further augmented by real-time microscopical assessment of tumor margins through CLE, enabling the surgeon to adapt their strategy of *marginal* resection on the fly. Albeit this appliance would be an extrapolation of the scope of our trial, Acerbi et al. elucidated the accuracy of CLE in this specific domain of tumor margin assessment in HGG surgery within their clinical trial (F. Acerbi, personal communication, June 30 2023).

A glaring deficit of CLE is found in its inability to detect or define any molecular pathological features, which in large part dictate decision-making in modern Oncology. The optical identification of light-tissue scattering fingerprints by Raman-based spectroscopy technologies has been of growing interest in the recent decade, and not only has one study demonstrated excellent diagnostic accuracy in a glioma mouse model as well as another in an in vivo series of 17 patients with mostly astrocytomas and glioblastomas, but the technique allows for detection of spectral bands specific to IDH1 mutations in situ with an accuracy of 89%.^37–39^ While the current application of Raman-based spectroscopy eschews a conventional histomorphological assessment, one investigation has leveraged *virtual* hematoxylin–eosin stains by mapping various wavelength profiles to unstained surgical specimens.^40^ This promising technique further eschews the need to label the specimen, that is, no fluorophore or staining agent is administered, which is a decisive advantage compared to CLE as it foregoes the risk of a false-negative labeling of regions with a low cancer cell burden and a false-positive labeling of pseudoprogressive disease in recurrent tumors. Unlike CLE, the technique’s effect is dependent on the energy applied; as such, the laser exposure must be tapered in an in vivo application to not risk damaging the tissue, thereby potentially weakening the interrogating effect of the scattered light.^41^ In principle, this promising technique offers a many-faceted portfolio of potential clinical applications that are expected to experience continuing adoption and commercialization, which, as for CLE, requires accompanying controlled trials investigating its validity and safety.

### Future Prospects

It is imperative that the implementation of a novel technique be stewarded by adequate training in its handling and assessment of CLE images. In addition to the installation of a telepathology consultation, a practical solution would see the establishment of a digital encyclopedia of CLE images, guided by case-based tutoring of hallmarks and pitfalls in CLE assessment. The technique lends itself well to such an approach, if only for its digital and atraumatic disposition. In a discussion of the potential of CLE for brain tumors, Maragkou et al. pointed out that a commonly specified assessment guideline and diagnostic criteria are quintessential, particularly in this early stage of technique adoption.^8^ Therefore, the analogous endeavors of other specialties that have already explored the utility of CLE have begotten diagnostic guidelines for Barrett’s esophageal dysplasia, cholangiocarcinoma, and urothelial carcinoma, reaching diagnostic accuracies of up to 89.7%.^42–44^ Owing to its readily available digitalized format with a high volume of assorted cumulative data, a deep-learning algorithm may provide an efficient computerized assessment of numerous intraoperative CLE images that are unspecific to the untrained human eye, seeing as one representative key feature image suffices for diagnosis.^45^ Consequently, Ziebart et al. successfully trained a neural network model to differentiate glioblastoma and metastasis tissue ex vivo.^46^

The technique’s quality and validity stand to improve over time, not only through the accumulation of expertise and academic scrutiny with its widespread adoption but also with the development of better contrasting agents. At present, CLE is used in conjunction with a dye that is rather unselective, as Fluorescein primarily stains *everything but* the cells, thereby enhancing background contrast. Promising new agents may be found in fluorophores bound to receptor-exclusive substrates, equipping CLE for a more specific detection of entities all the while possibly reducing its toxicity spectrum and reducing side effects after administration. The poly(ADP-ribose)polymerase biomarker has been investigated as a tracer for glioblastoma imaging, with promising results in preclinical human data, opening up its potential for glioblastoma-selective fluorescent cytoplasm staining with CLE.^47–50^

### Study Limitations

Given the lack of a homogenous representation of the various entities within our trial population, a subgroup comparison of the accuracies presented in our results should be avoided. In light of the fairly unselected population with a representation of tumor entities that is closer to real-life conditions, a verdict concerning the diagnostic accuracy, particularly for LGG, as well as reactive and inflammatory processes is not yet warranted. In concert with the still-accumulating experience with the technique and as the centrally conducted CLE assessments of one neuropathologist were opposed against the routine FS of the respective sites, our results could be biased *against* CLE. This design was deliberately chosen for our confirmatory trial investigating the validity of the technique without confounding by interrater discrepancies. We sought to amend this probable methodological deficiency post hoc through a blinded reassessment of all CLE images by 2 neuropathologists not involved with the initial study design, and have appended the results as Supplementary Material. While it becomes apparent that there is some disparity between assessments, more importantly, the post hoc assessments forfeited considerable diagnostic accuracy, which again brings to light that the validity and utility of the technique are products of the rater’s expertise, a dimension that is still in the process of being accumulated collectively and individually.

A technical limitation and significant methodological detriment of this trial stems from any juxtaposition of 2 microscopically assessed specimens, of which one is taken in vivo by CLE and the other *processed* in vitro for FS. We counteracted this with the aid of neuronavigation, although we cannot ascertain to have compared pools of precisely identical cells, rather identical regions of interest within the same lesion.

This study investigated the utility of CLE for the assessment of in vivo tissue by placing the CLE probe on a region of interest within the surgical site, which is unsuitable for analyses of specimens acquired by needle biopsy. While the CLE setup allows for mounting the CLE probe to the user station and thus facilitates the placement of such a biopsy specimen on the still probe for analysis, we did not include this use case within this study.

## Conclusions

In this first and to date largest clinical trial comparing the diagnostic validity of CLE with routine FS, we achieved an accuracy of 87% correct intraoperative diagnoses for CLE with administered Fluorescein compared with 91% for FS across a generalizable cohort of patients. The CLE technique was faster, with a 10-fold reduction of time expedited until an intraoperative diagnosis was achieved, allowing for real-time adaptation of the surgical strategy. Prospectively, this novel technique may accomplish higher diagnostic accuracy with continuing adoption and accrual of expertise.

## Supplementary Material

noae006_suppl_Supplementary_Data

noae006_suppl_Supplementary_Figures_S1

noae006_suppl_Supplementary_Tables_S1-S4

## Data Availability

The data presented herein will be available from the corresponding author upon reasonable request after publication. The findings of this study will also be made available on the European Clinical Trials Database under EudraCT identifier 2019-004512-58.
