# Supplementary material for: Fluorescein-stained confocal laser endomicroscopy versus conventional frozen section for intraoperative histopathological assessment of intracranial tumors
Source: Neuro Oncol. 2024 Jan 18;26(5):922–32. doi: 10.1093/neuonc/noae006 (PMC11066924; doi:10.1093/neuonc/noae006)
Supplement: noae006_suppl_Supplementary_Data [file noae006_suppl_supplementary_data.docx]

**Supplementary Table 1.** Overall inter-rater reliability. Sample data contains 203 effective subjects and 3 raters. Sig. – level of significance.

**Supplementary Table 2.** Inter-rater reliability stratified by histological entities. Sample data contains 203 effective subjects and 3 raters. Sig. – level of significance; category *Other* includes iron deposit, unspecified lesions and unknown entities.

**Supplementary Table 3.** Crosstable matrix of sensitivity and specificity for CLE evaluation of tumorous tissue versus non-tumorous tissue, referenced with the final histopathology. CLE – confocal laser endomicroscopy. Superscripts *a* and *b* denote values for specificity and sensitivity, respectively.

**Supplementary Table 4.** Adverse Events (AEs) listed by Organ Class and number of patients affected, multiple AEs per patient possible. n – number of patients. * - AE possibly related to CLE or Fluorescein.

**Supplementary Figure 1 A – C.** Intraoperative photographs of CLE probe in use with sterile draping (right hand). Note the similarity in shape and dimension to the surgical suction cannula.
