# Supplementary figures and images for: Fluorescein-stained confocal laser endomicroscopy versus conventional frozen section for intraoperative histopathological assessment of intracranial tumors
Source: Neuro Oncol. 2024 Jan 18;26(5):922–32. doi: 10.1093/neuonc/noae006 (PMC11066924; doi:10.1093/neuonc/noae006)

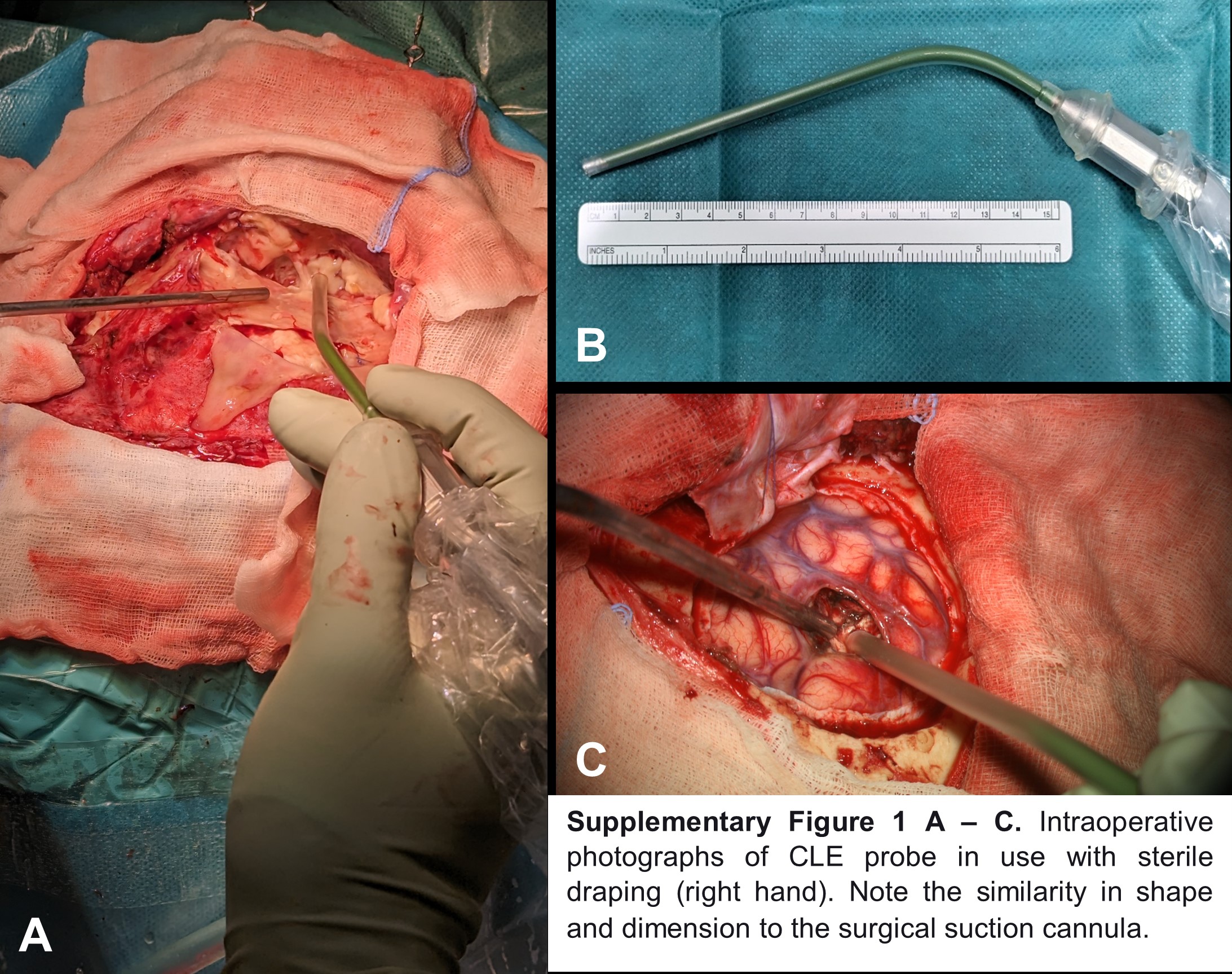

Supplement: noae006_suppl_Supplementary_Figures_S1 [file noae006_suppl_supplementary_figures_s1.jpeg]
