# Supplementary material for: Fluorescein-stained confocal laser endomicroscopy versus conventional frozen section for intraoperative histopathological assessment of intracranial tumors
Source: Neuro Oncol. 2024 Jan 18;26(5):922–32. doi: 10.1093/neuonc/noae006 (PMC11066924; doi:10.1093/neuonc/noae006)
Supplement: noae006_suppl_Supplementary_Tables_S1-S4 [file noae006_suppl_supplementary_tables_s1-s4.docx]

|  | **Kappa** | **Standard Error** | **Sig.** | **95% Confidence Interval** | |
| --- | --- | --- | --- | --- | --- |
|  |  |  |  | **Lower Bound** | **Upper Bound** |
| Overall Agreement | .872 | .023 | < .001 | .826 | .918 |
| **Supplementary Table 1.** Overall inter-rater reliability. Sample data contains 203 effective subjects and 3 raters. Sig. – level of significance. | | | | | |

| **Rating Category** | **Conditional Probability** | **Fleiss Kappa** | **Standard Error** | **Sig.** | **95% Confidence Interval** | |
| --- | --- | --- | --- | --- | --- | --- |
|  |  |  |  |  | **Lower Bound** | **Upper Bound** |
| HGG | .928 | .878 | .041 | .000 | .799 | .957 |
| LGG | .862 | .855 | .041 | .000 | .776 | .935 |
| Metastasis | .880 | .841 | .041 | .000 | .761 | .920 |
| Meningioma | .955 | .943 | .041 | .000 | .863 | 1.022 |
| Schwannoma | .714 | .708 | .041 | .000 | .628 | .787 |
| Ependymoma | 1.000 | 1.000 | .041 | .000 | .921 | 1.079 |
| Reactive | .333 | .330 | .041 | .000 | .251 | .409 |
| Inflammation | .333 | .330 | .041 | .000 | .251 | .409 |
| Other | .895 | .891 | .041 | .000 | .812 | .971 |
| **Supplementary Table 2.** Inter-rater reliability stratified by histological entities. Sample data contains 203 effective subjects and 3 raters. Sig. – level of significance; category *Other* includes iron deposit, unspecified lesions and unknown entities. | | | | | | |

|  | | | **Final Histopathology** | | **Total** |
| --- | --- | --- | --- | --- | --- |
|  |  |  | **No tumor** | **Tumor** |  |
| **CLE Assessment** | **No tumor** | Count | 7 | 2 | 9 |
|  |  | % within column | 43.8^a^ | 1.1 | 4.4 |
|  | **Tumor** | Count | 9 | 185 | 194 |
|  |  | % within column | 56.3 | 98.9^b^ | 95.6 |
| **Total** | | Count | 16 | 187 | 203 |
|  |  | % within column | 100.0 | 100.0 | 100.0 |
| **Supplementary Table 3.** Crosstable matrix of sensitivity and specificity for CLE evaluation of tumorous tissue versus non-tumorous tissue, referenced with the final histopathology. CLE – confocal laser endomicroscopy. Superscripts *a* and *b* denote values for specificity and sensitivity, respectively. | | | | | |

| **System Organ Class** | *Events* | *n* | *(%)* |
| --- | --- | --- | --- |
| **Eye disorders** | **15** | **13** | **(6)** |
| Eye swelling | 8 | 6 | (3) |
| Pupils unequal | 1 | 1 | (1) |
| Vision blurred | 1 | 1 | (1) |
| Visual impairment | 5 | 5 | (3) |
| **Gastrointestinal disorders** | **34** | **19** | **(9)** |
| Melaena | 1 | 1 | (1) |
| *Nausea | 22 | 12 | (6) |
| Swollen tongue | 2 | 1 | (1) |
| ***Vomiting | *11* | *5* | *(3)* |
| **General disorders and administration site conditions** | **2** | **2** | **(1)** |
| Pain | 1 | 1 | (1) |
| Swelling | 1 | 1 | (1) |
| **Infections and infestations** | **15** | **14** | **(7)** |
| Pneumonia | 6 | 6 | (3) |
| Post procedural infection | 1 | 1 | (1) |
| Urinary tract infection | 8 | 7 | (4) |
| **Investigations** | **6** | **6** | **(3)** |
| Blood pressure increased | 4 | 4 | (2) |
| Body temperature increased | 1 | 1 | (1) |
| Pupillary light reflex tests abnormal | 1 | 1 | (1) |
| **Musculoskeletal and connective tissue disorders** | **5** | **5** | **(3)** |
| Arthralgia | 1 | 1 | (1) |
| Back pain | 2 | 2 | (1) |
| Neck pain | 1 | 1 | (1) |
| Pain in extremity | 1 | 1 | (1) |
| **Nervous system disorders** | **40** | **21** | **(10)** |
| Aphasia | 1 | 1 | (1) |
| Brain oedema | 1 | 1 | (1) |
| *Dizziness | 5 | 3 | (2) |
| Facial paresis | 4 | 4 | (2) |
| ***Headache | *25* | *9* | *(4)* |
| Hemiparesis | 2 | 2 | (1) |
| Hypoaesthesia | 2 | 1 | (1) |
| **Psychiatric disorders** | **4** | **4** | **(2)** |
| Claustrophobia | 1 | 1 | (1) |
| Depressed mood | 3 | 3 | (2) |
| **Supplementary Table 4.** Adverse Events (AEs) listed by Organ Class and number of patients affected, multiple AEs per patient possible. n – number of patients. * - AE possible related to CLE or Fluorescein. | | | |
